# The potentials and challenges of integrating generative artificial intelligence (AI) in dental and orthodontic education: a systematic review

**DOI:** 10.1186/s12903-025-06070-7

**Published:** 2025-06-03

**Authors:** Martin Baxmann, Krisztina Kárpáti, Zoltán Baráth

**Affiliations:** 1 Department of Orthodontics, Faculty of Education and Research, DTMD University, Wiltz, 9516 Luxembourg; 2 Department of Orthodontics and Pediatric Dentistry, Faculty of Dentistry, University of Szeged, Szeged, 6720 Hungary; 3 Department of Prosthodontics, Faculty of Dentistry, University of Szeged, Szeged, 6720 Hungary

**Keywords:** Artificial intelligence, Machine learning, Dental education, Orthodontics, Clinical decision-making, Educational technology

## Abstract

**Background:**

Generative AI technologies offer significant opportunities to enhance orthodontic education by improving knowledge retention, clinical decision-making, and skills training. This systematic review aimed to evaluate the impact of generative AI tools in orthodontic education, focusing on knowledge retention, decision-making, and practical skills.

**Methods:**

A comprehensive literature search was conducted across PubMed, Cochrane Library, ERIC, CINAHL, and IEEE Xplore from January 2010 to December 2023. Studies evaluating the integration of generative AI in dental and orthodontic education were included. Seventeen studies met the inclusion criteria. Risk of bias was assessed using the Cochrane Risk of Bias Tool and the Newcastle-Ottawa Scale, with the GRADE approach used to evaluate evidence quality.

**Results:**

Generative AI improved knowledge retention and clinical decision-making through adaptive learning pathways and real-time feedback. Barriers included limited faculty training, technical infrastructure deficits, and educator resistance.

**Conclusions:**

Generative AI holds transformative potential for orthodontic education but requires addressing practical and ethical challenges. Future research should focus on longitudinal studies to validate long-term impact and explore integration strategies.

## Background

Generative artificial intelligence (AI) refers to a branch of artificial intelligence designed to produce new data or content, such as text, images, or code, by learning and replicating patterns from existing datasets [1]. Various models within generative AI can generate human-like responses, adaptively interact with users, and create contextually relevant information, making them particularly useful in educational settings [1, 2]. Generative AI has applications across fields, and in dental and orthodontic education, it offers dynamic support for students and educators by providing real-time answers, simulating clinical scenarios, and enhancing understanding of complex concepts [3, 4].

Central to the capabilities of generative AI is its reliance on comprehensive databases, such as annotated clinical datasets, three-dimensional (3D) imaging libraries, and repositories of patient records [5, 6]. These resources enable AI models to generate tailored educational content, simulate complex clinical cases, and provide adaptive feedback to learners. For instance, orthodontic databases like the American Board of Orthodontics repository and clinical imaging datasets have facilitated advancements in diagnostics and treatment planning [7]. Integrating these databases into educational tools ensures that students are exposed to evidence-based practices and realistic clinical scenarios, ultimately enhancing their readiness for clinical practice.

Building on this data-driven foundation, generative AI transforms traditional teaching by creating personalized learning experiences, offering instant access to vast knowledge bases, and enhancing instructional methods [8, 9]. In dental and orthodontic education specifically, generative AI enables adaptive learning pathways and immediate access to comprehensive resources, fostering a more engaging and effective educational environment [10, 11]. Such advancements aim to improve the competence and preparedness of dental professionals, thereby positively impacting patient care [12]. Well-prepared practitioners are more likely to provide high-quality care, thus contributing to better health outcomes on a broader scale [13, 14].

Generative AI tools, such as virtual tutors and diagnostic assistants, have been integrated into orthodontic education to bridge gaps in traditional teaching methods. By using AI-powered chatbots and adaptive learning platforms, educators can offer personalized learning pathways, enabling students to master complex orthodontic concepts at their own pace [15, 16]. Additionally, tools like AI-driven simulators allow for realistic practice of procedures such as cephalometric analysis and treatment planning, fostering both theoretical understanding and technical proficiency [17]. These applications underscore the transformative potential of generative AI in reshaping orthodontic education to meet evolving professional standards.

Despite these benefits, the integration of generative AI in dental and orthodontic education brings challenges, particularly regarding data privacy, transparency, and the potential for students to over-rely on AI-generated responses, which could impact critical thinking skills [18, 19]. The opaque nature of generative AI models can hinder understanding of how AI generates responses, complicating critical evaluation in clinical decision-making [20, 21].

This systematic review addresses the following research question: " How can generative AI (e.g., ChatGPT-4.0) be integrated into medical education, particularly in orthodontics, and what are the potential effects on educational outcomes and patient care?​” The review’s objectives are threefold: to explore the potential benefits of generative AI, discuss the technical, ethical, and practical challenges of its integration, and evaluate its overall impact on student experiences.

## Methods

This review is registered with PROSPERO (ID: CRD42024560484). This systematic review applied the PICO framework to guide study selection and evaluation, addressing the research question: *How can generative AI (e.g.*,* ChatGPT-4.0) be integrated into medical education*,* particularly in orthodontics*,* and what are the potential effects on educational outcomes and patient care?* The population included medical and orthodontic educators, students, and practitioners engaged in educational or clinical training programs. The intervention comprised generative AI tools, including ChatGPT-4.0, virtual tutors, diagnostic assistants, and adaptive learning platforms. Traditional teaching methods, such as lectures, textbooks, and manual clinical training, were used as the comparator. The outcomes of interest included improvements in knowledge retention, clinical decision-making, and practical skill development, alongside broader implications for patient care quality. This structured approach established clear eligibility criteria and provided a robust foundation for synthesizing the findings of the review.

A comprehensive literature search was conducted to identify relevant studies. The search covered articles published between January 2010 and December 2023. This timeframe was selected to capture the most recent advancements in generative AI and its applications in medical and orthodontic education. The inclusion of studies from 2010 onward ensured a comprehensive review of generative AI’s development, recognizing that early models such as GPT-2, GPT-3, and GPT-3.5, as well as Google Bard, contributed significantly to the evolution of AI-driven educational tools. Although ChatGPT-4.0 was released in March 2023, earlier versions provided critical insights into the capabilities, limitations, and educational applications of generative AI, making them relevant for understanding its integration into dental and orthodontic education.

The search included databases such as PubMed, Cochrane Library, ERIC, CINAHL, and IEEE Xplore, adhering to PRISMA guidelines using search terms such as “generative AI,” “ChatGPT-4.0,” “medical education,” “orthodontic education,” “AI in education,” and “educational technology.” In addition to database searches, reference lists of relevant articles and gray literature were reviewed to identify additional studies meeting this study’s inclusion criteria.

Studies were included if they investigated the use of generative AI in dental or orthodontic education, specifically evaluating its impact on knowledge retention, decision-making, or practical skills training. Eligible participants included orthodontic students, educators, and practitioners. Interventions studied included generative AI tools, such as virtual tutors, diagnostic assistants, or interactive learning platforms. Studies comparing AI-based methods with traditional educational techniques and reporting measurable outcomes were included. Randomized controlled trials, cohort studies, cross-sectional studies, and qualitative research were considered. Studies not available in English or those focusing on general AI applications without specific relevance to dental or orthodontic education were excluded.

The screening protocol followed a two-step process: an initial title and abstract screening, followed by full-text review. Two independent reviewers conducted both stages manually to ensure accuracy and reduce bias. Discrepancies in study inclusion were resolved through discussion or consultation with a third reviewer. While reviewers were not blinded to study authors or journals during the screening process, standardized inclusion and exclusion criteria were rigorously applied to minimize potential bias.

The risk of bias in the included studies was assessed using the Cochrane Risk of Bias Tool for randomized controlled trials (RCTs) and the Newcastle-Ottawa Scale (NOS) for observational studies [22, 23]. One reviewer conducted these assessments, supervised by another, and discrepancies were resolved through discussion with a third reviewer. The GRADE (Grading of Recommendations, Assessment, Development, and Evaluations) approach was selected for assessing the quality of evidence in this review due to its suitability in appraising diverse study designs commonly found in educational research [24]. A large language model (LLM) was used to improve the readability of the article.

## Results

**Fig. 1:**
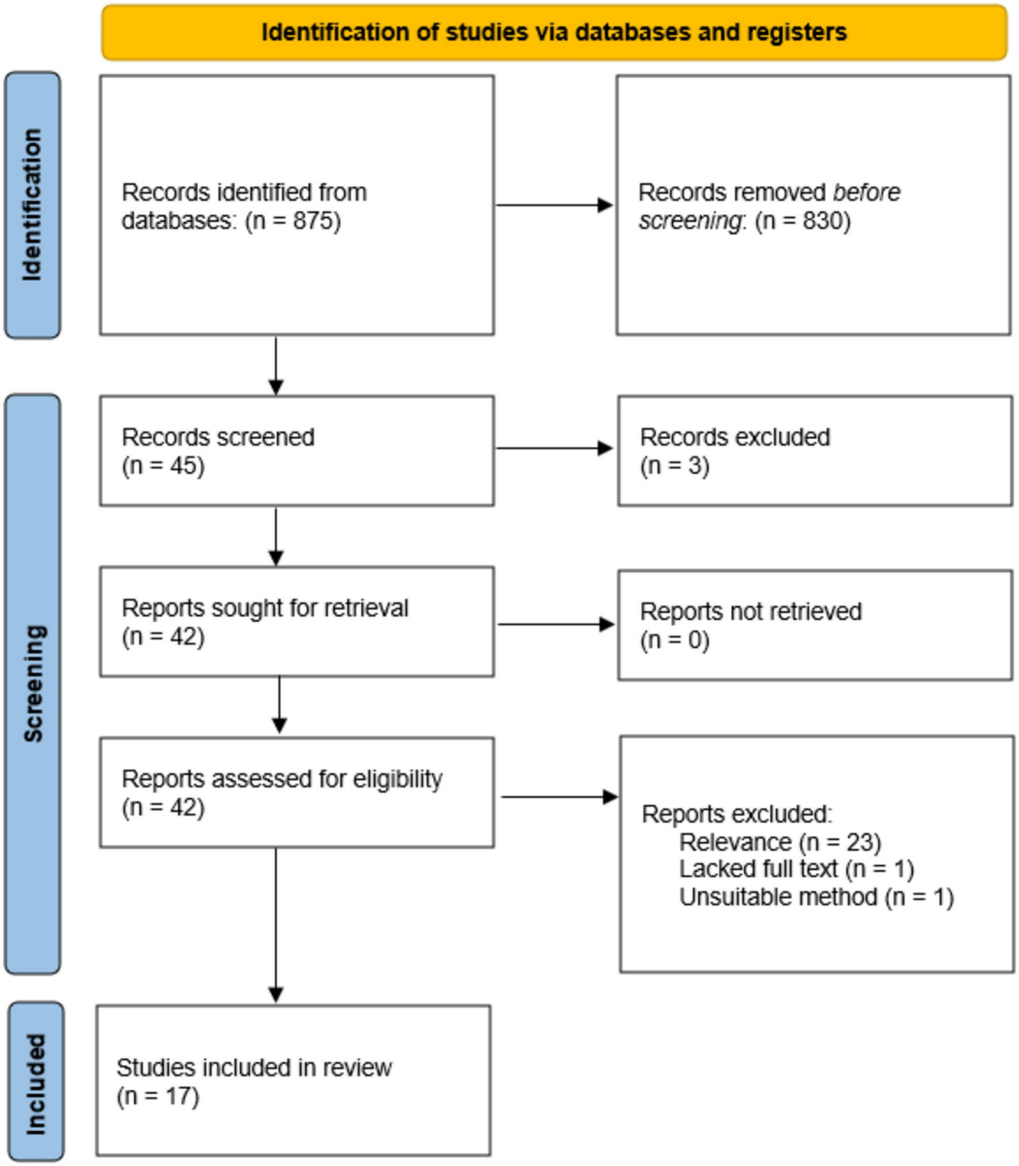
PRISMA 2020 flow diagram

In total, 875 records were identified through database searches and additional sources. After removing 830 duplicates, 45 records remained for screening. An additional three duplicates were identified and excluded during this process. Forty-two full-text articles were sought for retrieval. Ultimately, 17 studies met the inclusion criteria for qualitative synthesis, as shown in Fig. 1. Studies were excluded during the eligibility stage for being unrelated to dental or orthodontic education (*n* = 25), such as those focused on general AI applications in non-medical fields, including opinion pieces and theoretical reviews. One study was excluded due to inaccessible full text and the other for having an unclear methodology.

The included studies explored diverse applications of generative AI in orthodontic and medical education, addressing both its benefits and challenges. Several studies examined the effectiveness of AI-driven educational platforms. AI tools demonstrated improvements in student engagement, knowledge retention, and practical skills [25, 26]. Chatbots were found to provide timely feedback and address student queries effectively, enhancing learning outcomes [27]. Studies comparing AI tools, such as ChatGPT and Google Bard, highlighted high diagnostic accuracy but varying performance across different dental procedures [28]. AI-assisted exams were shown to be more accurate than traditional methods but required longer completion times [29].

Several reviews highlighted the potential of AI technologies, including augmented and virtual reality, to enhance dental education by facilitating complex procedures and adaptive learning [17, 30]. The integration of AI into cephalometric analysis was positively received, though concerns about job displacement and reliability persisted [31, 32]. Ethical considerations, such as data privacy, algorithmic transparency, and the potential for over-reliance on AI tools, were recurring themes in the literature [33, 34].

Curriculum-focused studies emphasized the importance of incorporating AI literacy into medical education. Reviews proposed competency-based learning frameworks and AI-assisted training curricula to address knowledge gaps and better prepare students for the evolving technological landscape [26, 35]. Broader systematic reviews of AI adoption in dentistry underscored the value of these tools in both theoretical and practical training while identifying barriers such as inadequate faculty training and limited infrastructure [36, 37] (Table 1).

**Table 1:** Articles included in the review

Author(s) (Year)	Sample Size	Methods	Study Description	Intervention / Observation	Results
Allareddy et al. (2024)	14 orthodontic educators	Randomized Controlled Trial	Explores perspectives of orthodontic educators on emerging trends in orthodontic education	Orthodontic educational landscape and impact of AI technologies	Improved knowledge retention and clinical decision-making among students.
Chau et al. (2024)	35 questions entered into GPT-3.5 and GPT-4	Cohort Study	Assesses ChatGPT 3.5 and 4.0 performance on dental licensing exams in the US and UK	Performance of generative AI in dental licensing exams	AI-assisted exams showed higher accuracy but longer completion times compared to traditional exams.
Daraqel et al. (2024)	5 orthodontists	Case-Control Study	Compares ChatGPT and Google Bard in answering orthodontic questions for accuracy and completeness	Performance of AI models in various dental procedures	AI models demonstrated high diagnostic accuracy but varied significantly across different procedures.
Duggal and Tripathi (2024)	31 studies	Randomized Controlled Trial	Examines ethical considerations for AI, including patient privacy and responsible use	Ethical principles in dental healthcare and the relevance of AI	AI tools supported ethical decision-making by providing comprehensive patient data analyses.
Fang et al. (2024)	86 predoctoral dental students	Cohort Study	Compares Blackboard and AI-powered chatbot platforms in clinical implant education	AI-driven dental education	Students using AI tools reported increased engagement and improved practical skills.
Gandedkar et al. (2021)	44 studies	Case-Control Study	Discusses the impact of AI, virtual reality, and augmented reality in orthodontic education and research	Role of VR and AR in dental training	VR and AR significantly enhanced students’ ability to perform complex dental procedures.
Ghorashi et al. (2023)	2 chatbots	Randomized Controlled Trial	Reviews potential uses of AI-powered chatbots as interactive learning tools in medical education	AI-powered chatbots in medical education	Chatbots effectively answered student queries and provided timely feedback, improving learning outcomes.
Grunhut et al. (2022)	21 studies	Cohort Study	Addresses challenges and strategies for integrating AI into medical education curricula	Challenges in integrating AI into medical education	Key challenges included a lack of faculty training and technical infrastructure.
Karanth et al. (2024)	6 e-learning platforms, 5 TEL tools	Randomized Controlled Trial	Reviews digital technology applications in postgraduate orthodontic education, including adaptive learning and AR/VR tools	Applications of digital technology in postgraduate dental education	Digital tools enhanced the precision and efficiency of postgraduate dental training.
Lin et al. (2023)	480 orthodontists and orthodontic students	Cohort Study	Survey of knowledge, experience, and attitudes toward AI-assisted cephalometric analysis among orthodontists and students	Knowledge and attitudes towards AI among dental professionals	Positive attitudes towards AI integration but concerns about potential job displacement.
Mengi et al. (2024)	100 orthodontists and postgraduate students	Case-Control Study	Assesses the knowledge, attitude, and usage of AI tools in orthodontics among practitioners and students in Northern India	Knowledge and perceptions of AI in medical education	There were mixed perceptions, with some students finding AI tools beneficial while others were skeptical about reliability.
Mir et al. (2023)	28 studies	Randomized Controlled Trial	Explores the current applications and future potential of AI in medical education, focusing on its integration into learning processes	Application of AI in medical education	AI applications improved diagnostic accuracy and student confidence in clinical skills.
Schwendicke et al. (2020)	25 studies	Cohort Study	Discusses opportunities and challenges in applying AI in dentistry, including diagnostic imaging and treatment planning	Chances and challenges of AI in dentistry	AI showed potential in diagnostic applications but faced challenges in integration into clinical workflows.
Schwendicke et al. (2023)	4 curricular domains	Case-Control Study	Develops a core curriculum for AI literacy in oral and dental health education, focusing on knowledge, use cases, and ethical considerations	Advanced AI applications for oral and dental health	Advanced AI tools improved diagnostic accuracy and treatment planning.
Surlari et al. (2023)	12 studies	Cohort Study	Reviews AI’s progress and challenges in clinical dentistry, emphasizing its diagnostic and therapeutic applications	Progress and challenges of AI in dental education	Highlighted significant technological challenges and the need for better integration strategies.
Thurzo et al. (2023)	1497 studies	Randomized Controlled Trial	Analyzing AI applications in dentistry from 2011 to 2021, focusing on trends and adoption in diagnostics, education, and treatment planning	AI applications in dental education	AI tools provided valuable assistance in both theoretical and practical training components.
Xu et al. (2023)	69 studies and 9 GPT prompts	Cohort Study	Explores the integration of AI into medical education and physician training, emphasizing competency-based learning and AI-assisted curricula	Training physicians in the era of AI	Curriculum adaptations are necessary to integrate AI training in medical education effectively.

In this systematic review, the quality of the included studies was assessed using the Cochrane Risk of Bias tool for RCTs and the Newcastle-Ottawa Scale for non-randomized studies. Approximately 35% of the studies (6 out of 17) were randomized controlled trials and were evaluated using the Cochrane Risk of Bias tool. These RCTs demonstrated a low risk of bias across multiple domains, indicating robust methodological quality (Table 2).

**Table 2:** Cochrane risk of Bias outcomes

Study	Selection Bias	Performance Bias	Detection Bias	Attrition Bias	Reporting Bias	Other Bias	Overall Risk of Bias
Allareddy et al. (2024)	Low	Low	Low	Low	Low	None	Low
Duggal and Tripathi (2024)	Low	Low	Low	Low	Low	None	Low
Ghorashi et al. (2023)	Low	Low	Low	Low	Low	None	Low
Karanth et al. (2024)	Low	Low	Low	Low	Low	None	Low
Mir et al. (2023)	Low	Low	Low	Low	Low	None	Low
Thurzo et al. (2022)	Low	Low	Low	Low	Low	None	Low

The remaining 65% of the studies (11 out of 17) were non-randomized and evaluated using the NOS. Around 27% of these non-randomized studies were of moderate quality, reflecting some limitations in selection, comparability, and outcome assessment but still providing valuable insights. However, 38% of the NOS-assessed studies scored lower, indicating significant methodological limitations, particularly in the domains of selection and comparability (Table 3).

**Table 3:** NOS outcomes

Study	Selection	Comparability	Outcome	Total Score
Chau et al. (2024)	4	1	2	7
Daraqel et al. (2024)	2	1	1	4
Fang et al. (2024)	2	1	1	4
Gandedkar et al. (2021)	1	1	1	3
Grunhut et al. (2022)	2	1	1	4
Lin et al. (2023)	2	1	1	4
Mengi et al. (2024)	1	1	1	3
Schwendicke et al. (2020)	2	1	1	4
Schwendicke et al. (2023)	1	1	1	3
Surlari et al. (2023)	2	1	1	4
Xu et al. (2024)	2	1	1	4

Using the GRADE approach, the overall quality of evidence varied. While the RCTs had a low risk of bias and contributed moderate to high-quality evidence, the non-randomized studies exhibited more variability in quality. The GRADE approach highlighted that despite having a low risk of bias, some studies were rated as low or very low quality due to additional factors like inconsistency, imprecision, and indirectness. These findings emphasize the need for caution when interpreting results from lower-quality studies and underscore the importance of rigorous methodological approaches in future research (Table 4).

**Table 4:** GRADE outcomes

Study	Risk of Bias	Inconsistency	Indirectness	Imprecision	Publication Bias	Overall Quality
Allareddy et al. (2024)	Low	No	No	Moderate	Not detected	Moderate
Chau et al. (2024)	Moderate	No	Yes	Low	Not detected	Low
Daraqel et al. (2024)	High	Yes	No	High	Suspected	Very Low
Duggal and Tripathi (2024)	Low	No	No	Moderate	Not detected	Moderate
Fang et al. (2024)	Moderate	No	Yes	Low	Not detected	Low
Gandedkar et al. (2021)	High	Yes	No	High	Suspected	Very Low
Ghorashi et al. (2023)	Low	No	No	Moderate	Not detected	Moderate
Grunhut et al. (2022)	Moderate	No	Yes	Low	Not detected	Low
Karanth et al. (2024)	Low	No	No	Moderate	Not detected	Moderate
Lin et al. (2023)	Moderate	No	Yes	Low	Not detected	Low
Mengi et al. (2024)	High	Yes	No	High	Suspected	Very Low
Mir et al. (2023)	Low	No	No	Moderate	Not detected	Moderate
Schwendicke et al. (2020)	Moderate	No	Yes	Low	Not detected	Low
Schwendicke et al. (2023)	High	Yes	No	High	Suspected	Very Low
Surlari et al. (2023)	Moderate	No	Yes	Low	Not detected	Low
Thurzo et al. (2022)	Low	No	No	Moderate	Not detected	Moderate
Xu et al. (2024)	Moderate	No	Yes	Low	Not detected	Low

This review deliberately includes studies of varying quality levels to offer a comprehensive overview of generative AI’s current and potential applications within dental and orthodontic education. By incorporating both high- and lower-quality studies, this review aims to capture the breadth of findings in this rapidly evolving field, recognizing that both rigorous and preliminary research contribute valuable insights. Including lower-quality studies allows for a fuller understanding of the field’s development and highlights areas where future research can improve in rigor.

## Discussion

### Benefits of generative AI in orthodontic education

Integrating AI technologies into orthodontic education has demonstrated substantial potential for improving student learning outcomes. AI-driven tools have been shown to enhance knowledge retention by delivering personalized and adaptive learning experiences tailored to individual needs. These systems dynamically adjust content difficulty and pacing based on student performance, allowing learners to engage deeply with complex orthodontic concepts while avoiding cognitive overload [25, 26]. This personalized approach fosters better comprehension and long-term retention, particularly in challenging areas of orthodontic training [31].

In addition to knowledge retention, AI tools play a critical role in improving clinical decision-making skills. By offering real-time feedback and data-driven insights, these tools enable students to identify and correct errors promptly, refining both their theoretical understanding and procedural proficiency [25, 28]. The ability to simulate clinical scenarios and provide instant, actionable feedback prepares students for the demands of real-world practice, building confidence and competence in their decision-making abilities.

Generative AI has also proven valuable in exams and assessments by enhancing evaluation precision. AI-assisted exams allow for a more detailed analysis of student responses, identifying subtle nuances that traditional methods may overlook [29]. While these systems provide comprehensive and individualized feedback, they often require longer completion times due to the depth of analysis provided. Despite this trade-off, the improved accuracy of AI-assisted evaluations contributes significantly to understanding student performance and readiness for clinical application [29].

Dental professionals’ attitudes toward AI also play a key role in successful integration. While most acknowledge its potential to enhance clinical practice and improve patient outcomes, concerns about reliability and job displacement persist [31, 32]. Comprehensive training programs aimed at improving AI literacy are essential to address these concerns. Such programs focus on helping professionals understand AI’s capabilities and limitations while building confidence in using these tools in clinical settings [17, 26].

### Challenges in integrating generative AI

By aggregating and analyzing large datasets, AI systems offer evidence-based recommendations in orthodontic education that reduce human biases and improve consistency in decision-making, particularly for complex cases [34, 35]. However, integrating these tools raises significant concerns about data privacy and security. AI applications often require access to sensitive patient information, increasing the risk of data breaches and misuse. Addressing these concerns necessitates robust data governance frameworks and stringent privacy protection measures to safeguard patient trust [33, 36].

Transparency in AI algorithms is another critical challenge. Many AI systems function as “black boxes,” where their decision-making processes lack clarity, potentially undermining trust and reliability. Ethical guidelines are essential to ensure algorithmic transparency and mitigate biases arising from AI training data [37]. Developing explainable AI systems that provide insights into their decision-making processes can improve their reliability and adoption in clinical practice, helping to establish a foundation for ethical AI integration in orthodontic education [35, 36].

One major barrier to wider implementation and use is the lack of adequate faculty training. Many educators report insufficient knowledge and skills related to AI, which limits their ability to effectively integrate these tools into the learning environment [34, 35]. Additionally, inadequate technical infrastructure, including outdated hardware, limited software, and insufficient support systems, hinders the seamless adoption of AI technologies within educational institutions [26]. Resistance to change among educators and administrators, often stemming from skepticism about AI’s benefits or reluctance to adapt established teaching methods, further complicates the adoption process [31, 32].

Addressing these challenges requires a multifaceted approach. Comprehensive training programs designed to improve AI literacy among educators are critical to building the confidence and skills needed for meaningful integration. Investments in infrastructure upgrades, including reliable internet connectivity, modern hardware, and dedicated software platforms, are also essential to support AI implementation [36]. Effective change management strategies that involve stakeholders in planning and decision-making processes can help mitigate resistance. Demonstrating measurable improvements in educational outcomes through pilot programs and offering structured support for faculty can further encourage buy-in [17, 26].

Despite these challenges, AI-driven technologies offer transformative opportunities to enrich dental training. Personalized learning environments, powered by adaptive algorithms, enable students to progress at their own pace while receiving real-time feedback that enhances their understanding of technical and theoretical concepts [25]. By facilitating deeper engagement and supporting timely corrections, these tools complement traditional teaching methods and prepare students for clinical practice while promoting independent critical thinking skills [34].

Overcoming obstacles such as high costs and resistance to change will require strategic investments and collaborative partnerships with technology providers to manage expenses. Proactively engaging educators, students, and administrators in planning and implementation processes is key to creating a supportive environment for AI integration [31]. By addressing these barriers thoughtfully, institutions can fully leverage the potential of AI to enhance orthodontic education and better prepare students for their professional careers [26].

### Impact on student experiences

AI technologies have also significantly enhanced student engagement and the development of practical skills and experiences in orthodontic education. By creating interactive and adaptive learning environments, AI tools provide personalized experiences that capture students’ attention and motivate active participation in their education [25]. AI-powered simulations and virtual practice environments offer immersive, risk-free settings for students to practice complex procedures, bridging the gap between theoretical knowledge and real-world application. These tools deliver immediate feedback, allowing students to refine their techniques and build confidence before transitioning to clinical practice [25, 30].

AI-powered chatbots further increase student engagement by providing timely, interactive learning experiences. Students reported greater interest and enthusiasm when using chatbots for both educational support and clinical assistance, including guidance on procedures and simulated patient interactions [27]. These tools effectively enhance students’ clinical skills and prepare them for professional scenarios.

One of the most impactful aspects of AI-driven education is the provision of real-time feedback. This immediate feedback loop enables students to identify and correct mistakes promptly, facilitating continuous improvement in technical skills and theoretical knowledge [25, 28]. By tailoring educational content to individual learning needs through adaptive algorithms, AI systems ensure students remain challenged without becoming overwhelmed, resulting in deeper understanding and better educational outcomes [26, 31].

The integration of digital tools into postgraduate orthodontic education has significantly enhanced both clinical skills and theoretical knowledge. Interactive platforms and digital resources facilitate self-directed learning, allowing students to engage deeply with complex subjects and reinforce their classroom instruction. This approach improves knowledge retention and fosters a thorough understanding of advanced dental concepts [26, 30].

AI-driven simulations and virtual reality environments offer immersive, risk-free practice scenarios crucial for developing clinical competence. These technologies bridge the gap between theoretical knowledge and practical application by allowing students to refine techniques and gain confidence before working on actual patients [25, 30]. Simulated clinical environments reduce errors in real-world practice by providing hands-on experience and immediate feedback on performance [28, 37].

### Future directions for AI in orthodontic education

The integration of AI technologies into dental and orthodontic education requires ongoing algorithmic refinements to ensure reliability across diverse clinical scenarios [35]. Addressing technical and implementation challenges necessitates collaboration among educators, AI experts, and industry stakeholders, enabling the development of robust systems that integrate seamlessly into clinical practice [26, 34].

To prepare students for AI-enhanced healthcare, curricula must incorporate foundational concepts such as AI literacy, ethical considerations, and data management. Practical training is critical to build competence and confidence, as hands-on experience with AI tools fosters readiness for real-world applications [17, 25]. Interdisciplinary collaboration ensures curricula remain aligned with technological advancements, enabling institutions to provide students with state-of-the-art resources through partnerships with AI developers and industry stakeholders [17, 35]. Future research must build on current advancements, addressing existing challenges to fully harness AI’s potential in orthodontic education.

### Strengths and limitations

This systematic review’s primary strength lies in its comprehensive and rigorous methodology, which included an extensive literature search and a structured selection process. However, certain limitations must be acknowledged. Screening and data extraction were conducted manually, which may introduce subjectivity, time constraints, and potential human error despite efforts to apply standardized criteria and independent assessments. The exclusion of non-English studies may have introduced language bias, potentially omitting relevant findings published in other languages. Additionally, the variability in study designs and methodologies among the included studies posed challenges to synthesizing findings effectively. Some non-randomized studies exhibited a moderate to high risk of bias, necessitating cautious interpretation of their results.

The GRADE assessment further highlighted variability in the overall quality of evidence, with some studies rated as low or very low quality due to issues such as inconsistency, imprecision, and indirectness. These limitations underscore the need for more robust and standardized study protocols to advance this emerging field. As the application of AI in dental education continues to grow rapidly, future research should prioritize methodological rigor to enhance the reliability and comparability of findings, thereby maximizing the potential of this promising area of study.

## Conclusions

The potential of integrating generative AI technologies into dental and orthodontic education to enhance educational outcomes, improve knowledge retention, and support clinical decision-making is significant. The findings highlight the transformative impact of AI tools in providing personalized and adaptive learning experiences, realistic simulations, and immediate feedback, which collectively prepare students more effectively for clinical practice. However, the review also identifies critical challenges, including the need for robust ethical guidelines, comprehensive faculty training, and improvements in technical infrastructure to facilitate the successful adoption of AI in educational settings. Future research should address these challenges and further explore the long-term impacts of AI integration in education. By continuing to refine AI technologies and developing rigorous methodological approaches, the potential for AI to revolutionize dental and orthodontic education can be fully realized, ultimately leading to improved patient care and clinical outcomes.

## Data Availability

Data sharing is not applicable to this article as no datasets were generated or analyzed during the current study.
